# Effect of the Anisotropy Mechanical Properties on LN Crystals Fixed-Abrasive Lapping

**DOI:** 10.3390/ma13194455

**Published:** 2020-10-08

**Authors:** Nannan Zhu, Jiapeng Chen, Piao Zhou, Yongwei Zhu

**Affiliations:** 1 Engineering Technology Training Center, Nanjing Vocational University of Industry Technology, Nanjing 210046, China; 2 Jiangsu Key Laboratory of Precision and Micro-Manufacturing Technology, Nanjing University of Aeronautics and Astronautics, Nanjing 210016, China; 15937311032@163.com (J.C.); zhou2669piao@163.com (P.Z.); meeywzhu@nuaa.edu.cn (Y.Z.)

**Keywords:** lithium niobate crystals, anisotropy, nanoindentation, nanoscratching, critical cutting depth, fixed-abrasive lapping

## Abstract

The anisotropy of lithium niobate (LN) single crystals in mechanical properties affects its material removal uniformity during lapping. The nano-indentation hardness (*H*_I_) and elastic modulus(*E*) of Z-cut wafer and X-cut wafer were measured by a nano-indentation tester. The nano-scratching tests were adopted to evaluate its critical cutting depth (*d_c_*) of brittle ductile transition along crucial orientations of Z-cut and X-cut, respectively. A series of fixed-abrasive lapping tests were carried out to explore the effect of anisotropy on the lapping process. The results indicated that the *H*_I_ of Z-cut was slightly higher than that of X-cut, while the *E* of Z-cut was about 1.1 times of the latter. The *d_c_* value of each orientation varies greatly. The lapping tests showed that the material removal rate (MRR) of Z-cut was lower than that of X-cut, for its high *H*_I_ and *E*. Meanwhile, the surface quality of Z-cut was better than that of X-cut, for the larger *d_c_* of Z-cut. The research of mechanical properties of LN has guiding significance for its lapping process.

## 1. Introduction

Lithium niobate (LN) crystal is one of multi-functional materials which owns piezoelectric, ferroelectric, pyroelectric, nonlinear, lightning, and photorefractive properties, together with a good thermal stability and chemical stability. It has been applied on the surface acoustic wave filter, optical waveguide substrate, and infrared detectors, etc. [1,2]. Ultra-precision machined surfaces is the prerequisite for the application of LN crystals in each field. However, LN crystal is a typical strong anisotropy soft-brittle crystal (mohs hardness 5), with a thermal expansion coefficient of the a axis being more than eight times that of c axis (16.7 × 10^−6^ of a axis and 2.0 × 10^−6^ of c axis, respectively), and with different optical performance on each plane [3,4]. The difference in mechanical properties on different planes and orientations reduces the controllability of ultra-precision processes (here mainly refers to lapping and polishing process). In order to realize the ultra-precision machining of LN crystal, the research of the influence of anisotropy mechanical properties on the machining process is indispensable.

The anisotropy of materials has always been great concern. Naumov [5] provided a detailed overview of the current state of the emerging field of mechanically responsive single crystals. A general model for the mechanical effects in molecular crystals had been established in this work. An approach has been proposed to describe and explain mechanical and photomechanical phenomena at semiquantitative and quantitative levels. Arkhipov [6] found that the plasticity of L-Leucinium hydrogen maleate crystals can be preserved at least down to 77 K. The structural changing in the temperature range 293–100 K were followed in order to rationalize the large anisotropic plasticity in this organic compound. The “sliding layers” mechanism was used to describe the high plasticity of L-Leucinium hydrogen maleate in Nguyen’s work [7]. Moreover, one principle direction for sliding and corresponding bending was suggested, demonstrating only low-energy vdW interactions. The anisotropic has also been studied in semiconductor materials [8], such as *t*-Si_3_N_4_, *t*-Si_2_GeN_4_, *t*-SiGe_2_N_4_, and *t*-Ge_3_N_4_. In Han’s work, the elastic anisotropic of these materials was investigated. The elastic modulus decreased with the increasing of the proportion of Ge. The *t*-Ge_3_N_4_ showed the largest anisotropy among the *t*-Si_x_Ge_3-x_N_4_ (x = 0,1,2,3) alloys.

Some researches on the crystal anisotropy in mechanical properties have been reported. With the aid of nano-scratching, the study of friction, wear, and plastic deformation on KDP crystals indicated that the fracture toughness along [110] orientation was relatively low [9]. The morphological anisotropy of the machined surfaces was analyzed by the two-dimensional PSD method in Chen’s study [10]. The tangential waves along orientation of the machined surfaces were analyzed using wavelet-transform and PSD methods. The machined surface of the KDP workpiece exhibited strong anisotropy, and the features of the surface were mainly composed of contours in the cutting directions. When the indentation and scratching tests applied to HgCdTe crystal [11], the *E* was approximately at 50 GPa with the indentation depth varying from 200 to 800 nm, and the friction coefficient was retained basically constant. Size effect was observed on the nanohardness, which was addressed by indentation curvature. Zhang et al. [12] indicated that the (111) plane of Cd_0.9_Zn_0.1_Te was more fragile, and easily brought about serious microscratches by a nano-scratching test on CdZnTe crystals, while the shear bands appeared on the (111) of Cd_0.96_Zn_0.04_Te. Nano-indentation tests were conducted on LN crystals [13], focused on the pop-in phenomenon during loading, and the results showed various hardness on different planes. Although the nano-scratching and nano-indentation tests have been gradually adopted in material mechanical properties evaluation to guide the design of process parameters [14,15,16], the study on LN is still very rare, especially on the critical cutting depth along specific orientations.

In this study, the *H*_I_ and *E* of soft-brittle LN crystals will be measured by the nano-indentation tester. The critical cutting depth *d*_c_ of brittle ductile transition will be obtained to explore the most liable plastic deformation orientation. Furthermore, lapping tests were carried out, with the anisotropy characteristic to select the processing orientation. The micro/nano-scale mechanical properties of LN are useful to the parameter determination in the lapping process.

## 2. Materials and Methods

### 2.1. Specimen Preparation

The specimens in this study were cut from Z-cut and X-cut with a diameter of 75 mm and 1 mm in thickness. Prior to nano-indentation and nano-scratching, all wafers should be lapped and polished to be ultra-smooth on both surfaces and damage-free. For high efficiency, a fixed abrasive pad [17,18,19,20,21] was adopted in lapping processing. Thereby, 50 wt.% of W14 diamond grits (grain size of 10–14 μm) were fixed into this lapping pad, and the slurry was the deionized water only mixed with 0.2% OP-10. The lapping parameters were: applied load of 15 kPa and table platen of 80 rpm. The average surface roughness (Ra) of lapped wafer was about 10 nm by AFM. After that, followed by 30 min’s polishing on a polyurethane pad. The solution with a diameter of 90–100 nm silica was chosen as the slurry, with a pH around 8.5. The applied load was 10.5 kPa and the platen speed 60 rpm. The Ra of polished surface was less than 1 nm. After polishing, each wafer was cut into 15 × 10 mm patches. The specimens were subject to corrosion in 2% hydrofluoric acid solution for 2 min for stress-relieving, and then ultrasonic cleaning and drying for testing.

### 2.2. Nano-Indentation and Nano-Scratching Tests

**Figure 1:**
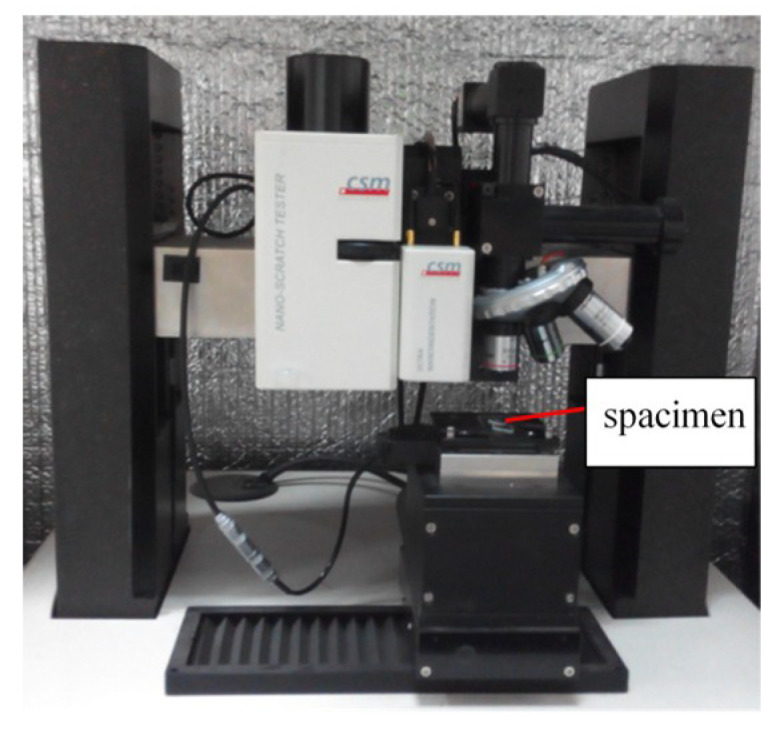
CSM Tribo-Indenter and the specimen.

**Figure 2:**
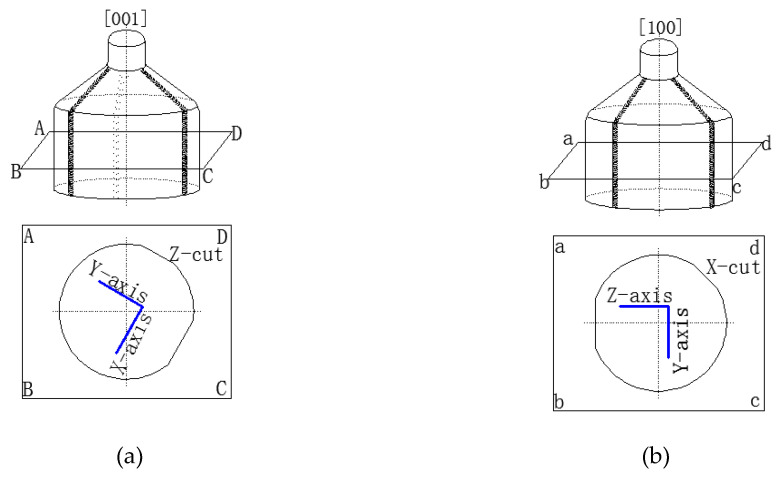
Scratching directions on LN different crystal wafers: (**a**) Z-cut, (**b**) X-cut.

**Figure 3:**
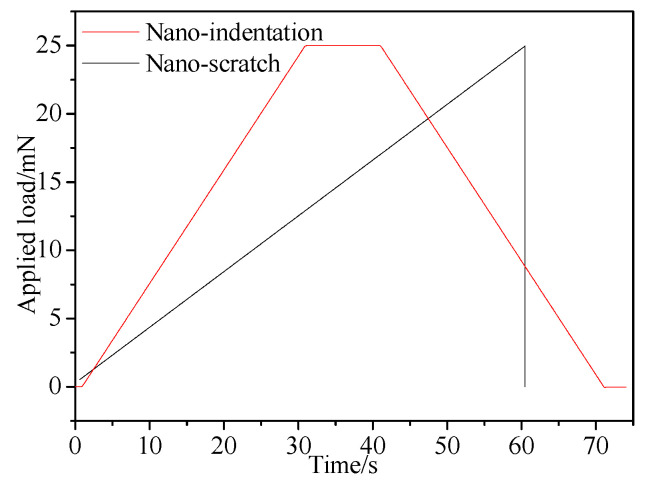
Loading-unloading curves of nano-indentation and nano-scratching.

Both nano-indentation and nano-scratching tests were conducted on a CSM Tribo-Indenter at room temperature, as shown in Figure 1. An in situ imaging system was incorporated to the CSM tester to examine the testing area topography after indentation and scratching. During the nano-indentation process, the diamond Berkovich indenter tip (3-side pyramid) penetrated each specimen at a constant speed of 50 nm/min, reached the pre-set maximum *F_m_* (25 mN), held for 10 s, and then unloaded. The average value of the 5 nano-indentation points was taken for each plane. When scratching along the specific orientations (shown in Figure 2), an indenter with a tip radius of 2.0 μm was selected. The whole test process consisted of three steps. Firstly, the specimen surface was pre-scanned with a load of 0.1 mN to obtain its primary topography. This was followed by the scratching step, in which the normal applied load linearly increased from 0.5 mN to maximum 25 mN at a constant scratching speed of 0.2 mm/min, and each scratch was 200 μm long. During scratching, the depth sensitive system detected the indenter’s vertical indentation depth real-timely and the depth-displacement curve and friction-load curve were obtained. After that, the surface morphology after elastic recovery was post-scanned by in situ imaging system. The loading unloading parameters for both nano-indentation and nano-scratching are shown in Figure 3.

### 2.3. Fixed-Abrasive Lapping Tests

**Figure 4:**
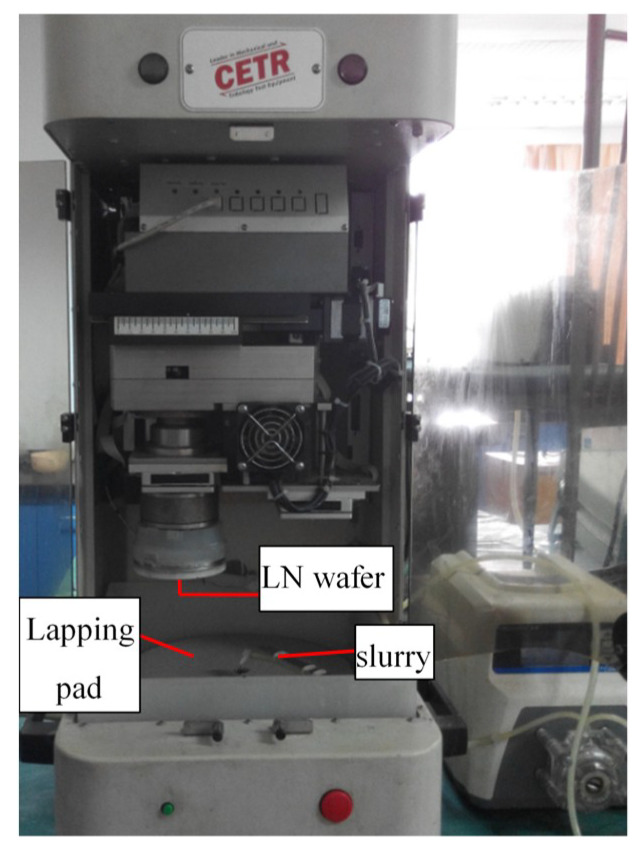
Fixed-abrasive lapping tests with CP-4 test system.

A series of fixed-abrasive lapping tests were carried out to explore the effects of the anisotropy of LN wafers on the lapping process. The specimens were Z-cut and X-cut LN wafers, with the Ra between 40 and 50 nm, a diameter of 70 mm, and thickness of 0.5 mm. All tests were carried out on the CP-4 test system with a pad fixed with W14 diamond abrasives in the hydrophilic resin matrix, as shown in Figure 4. The slurry was mainly deionized water added to 0.2% OP-10 emulsifier with a flow rate of 100 mL/min, for improving the wetness of the lapping pad. The lapping conditions were uniformly as follows: the applied load was 15 kPa, the table platen was 80 rpm, the eccentricity was 60 mm, and each lapping test lasted for 20 min. Before each test, the pad should be finished by whetstone for 1 min. After the test, the wafers were cleaned by ultrasonic and dried for detection. The surface morphology was observed by Leica microscope, the Ra was measured by AFM (Bruck, Germany), and the material removal rate (MRR) of each wafer was calculated.

## 3. Results and Discussions

### 3.1. Nano-Indentation Hardness H_I_ and Elastic Modulus E

**Figure 5:**
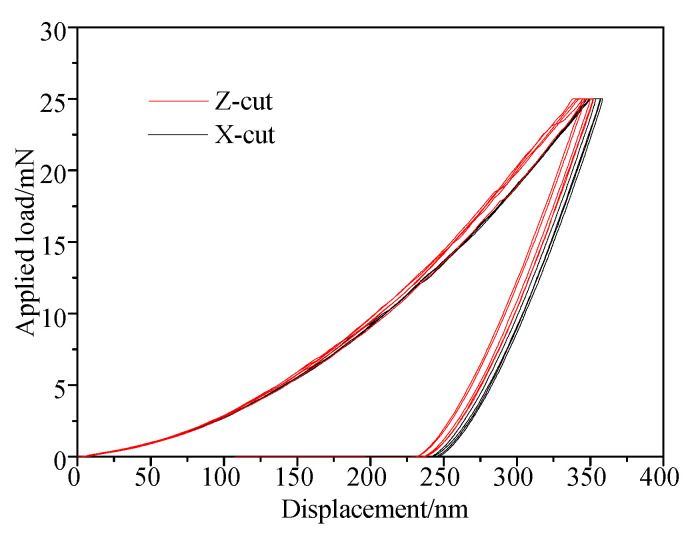
Applied load-displacement curves of Nano-indentation tests.

**Figure 6:**
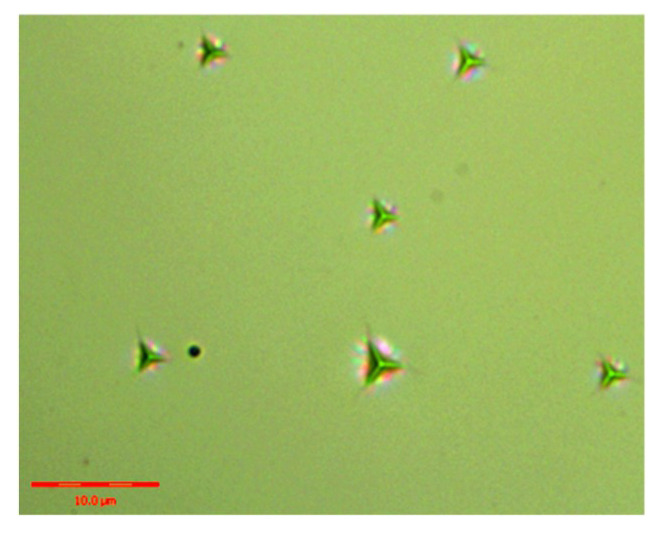
The indentation morphology by nano-indentation experiments.

**Figure 7:**
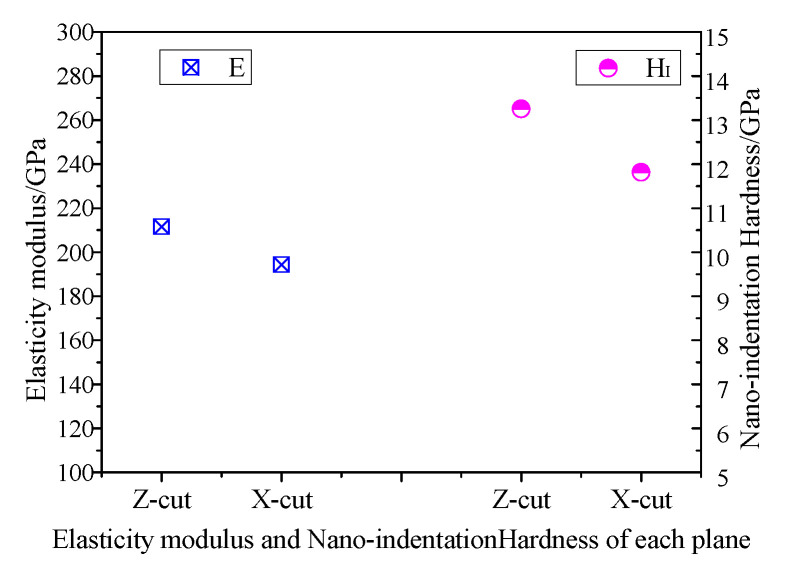
Elastic modulus and nano-indentation Hardness of LN.

Figure 5 shows the applied load-displacement curves of Z-cut and X-cut nano-indentation experiments. The maximum depth of indenter, *h_max_*, was obtained when applied load reached the 25 mN with a holding time of 10 s. During unloading, the indentation depth is affected by material elastic recovery. The nano-indentation morphology is shown in Figure 6. The plastic deformation became permanent and the the *H*_I_ and the *E* were calculated in accordance with the residual depth. Figure 7 shows that the *H*_I_ and *E* of X-cut are lower than that of Z-cut. The *H*_I_ and *E* of X-cut are 11.81 GPa and 194.3 GPa, respectively. Meanwhile, the *H*_I_ and *E* of Z-cut are 13.25 GPa and 211.5 GPa.

**Figure 8:**
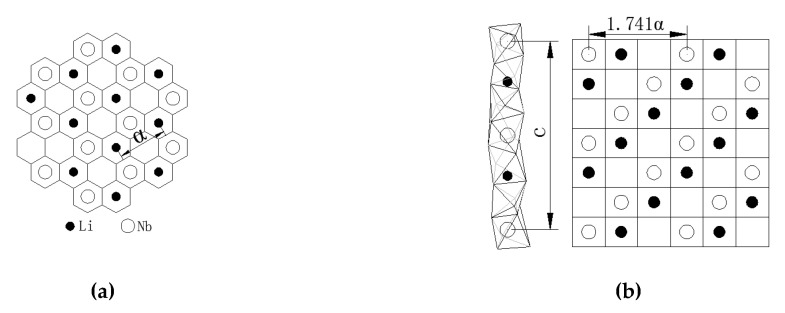
Schematic illustration of atomic structure of LN:(**a**) Z-cut, (**b**) X-cut

Elastic modulus evaluates the property of deformation resistance in the macro-scale, and can be regarded as the bonding strength between atoms in the micro-scale. The greater *E* is, the greater the stress to force material to deform should be. The factors affecting bonding strength will also affect the material elastic modulus, such as valence-bond type, crystal structure, chemical composition, micro-structure, and temperature, etc. The skeleton of a single crystal LN is composed of oxygen octahedrons. The layer structure perpendicular to Z axis presents hexagonal arrangement (Figure 8a). Nb atoms and Li atoms fill these octahedrons alternately. Along the Z axis, adjacent oxygen octahedrons share a public plane to connect into a series of distorted oxygen octahedral columns [3]. Nb atoms and Li atoms fill periodically in the Nb-Li-vacancy structure. The Z-cut wafer and X-cut wafer marked in Figure 8 indicate that atoms on the Z-cut arrange closely and the atomic spacing is a little smaller than that of X-cut. Therefore, inter-atomic bonding on Z-cut is stronger and the deformation resistance is also greater, resulting in its elastic modulus *E* and nano-hardness *H_I_* both being slightly higher than those of X-cut.

### 3.2. Critical Cutting Depth d_c_ of Brittle-Ductile Transition on Each Orientation

**Figure 9:**
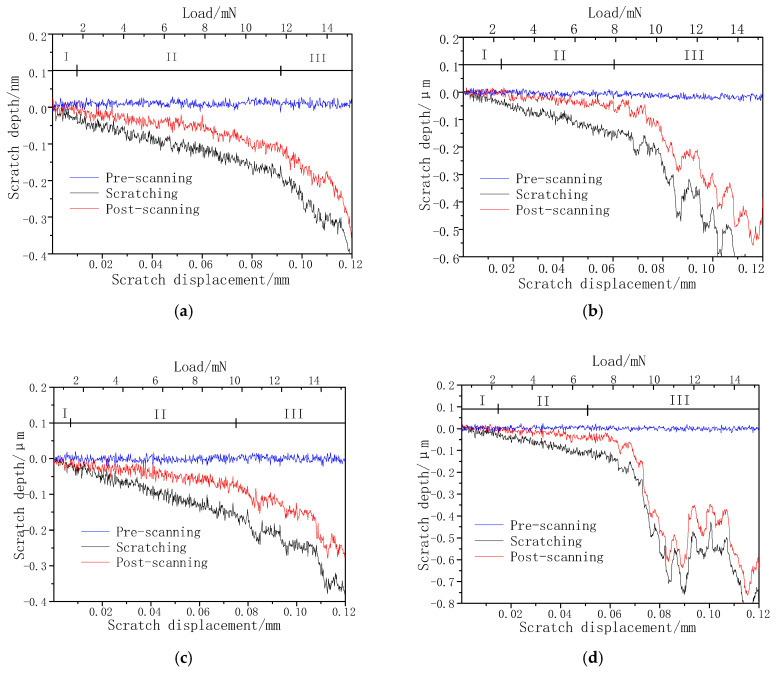
Scratch depth-displacement-load along different orientations: (**a**) X axis on Z-cut, (**b**) Y axis on Z-cut, (**c**) Y axis on X-cut, (**d**) Z axis on X-cut, of pre-scratching (I represents the elastic deformation stage, II represents the ductile deformation stage, and III represents the brittle deformation stage.).

The pressure depth displacement load curves of each orientation scratching test are shown in Figure 9a–d. This study focused on the transformation from the ductile deformation stage to the brittle deformation stage, estimating the transformation displacement through the curve slope fluctuation combining with the micromorphology of the scratches [22].

As shown in Figure 9, the curves of the four orientations differ obviously. Before and after scratching, the curves of X axis on Z-cut were relatively neat, and the distinct slope change appears as the indenter displacement slowly increased to 90 μm, which means the wiped removal on sample surface can be ascribed to plastic removal before 90 μm. Meanwhile, the depth displacement curve along the other orientation on Z-cut fluctuates severely before 60 μm. The same curve of the Y axis on X-cut fluctuates slightly smoothly and the slope changes near 75 μm, which indicates that the brittle ductile transition displacement of this orientation is increased than the former. The most significant fluctuation of curves happen on the scratching curves of the Z axis on X-cut, where cracks arise around 45 μm.

**Figure 10:**
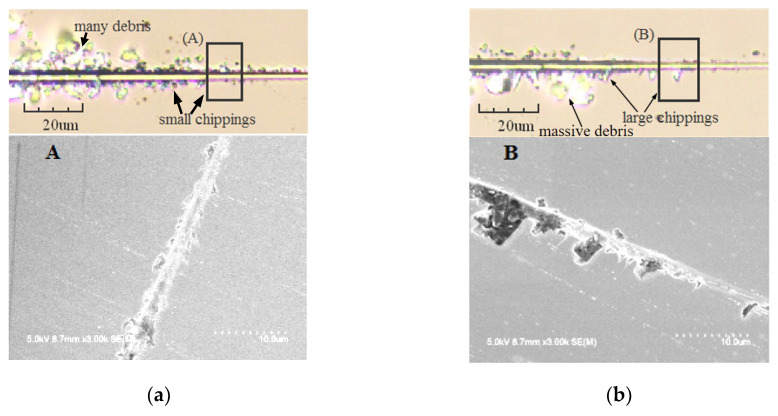

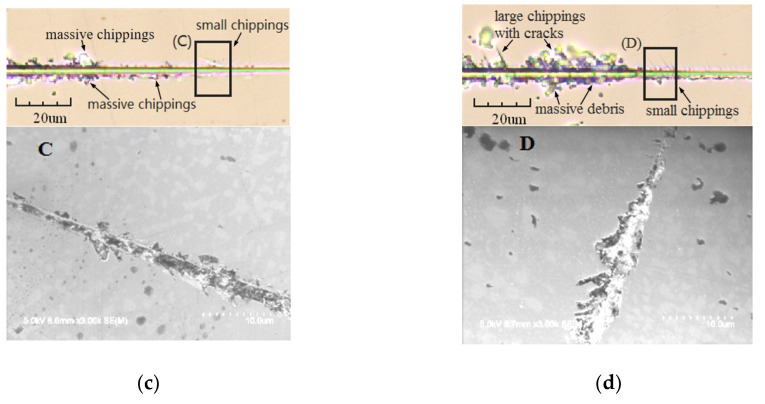
Scratches in suit scanning and SEM part magnification: (**a**) X axis on Z-cut, (**b**) Y axis on Z-cut, (**c**) Y axis on X-cut, (**d**) Z axis on X-cut.

Combined with Figure 10 (SEM of the scratches), the critical cutting depth *d*_c_ and critical applied load of brittle-ductile transition along each orientation are listed in Table 1. It can be seen that for the Z-cut, the *d*_c_ of X-axis is larger than that of Y-axis, and the *d*_c_ of Z axis on X-cut is the minimum. As for the *d*_c_ of Y axis on different planes, it is less on the Z-cut than that on X-cut.

LN anisotropy has a close relationship with the structure of the crystal itself. Due to the different atom arrangement of different planes and orientations, *E* and *d*_c_ measured on each orientation vary considerably. LN crystals structure belongs to a trigonal system and 3m lattice group. A hexagonal original cell is the commonly used cell expression, similar to the hexagonal close-packed structure. The (001) plane (the Z-cut) acts as the slip plane and the (100) direction (X axis) as the slip orientation.

**Figure 11:**
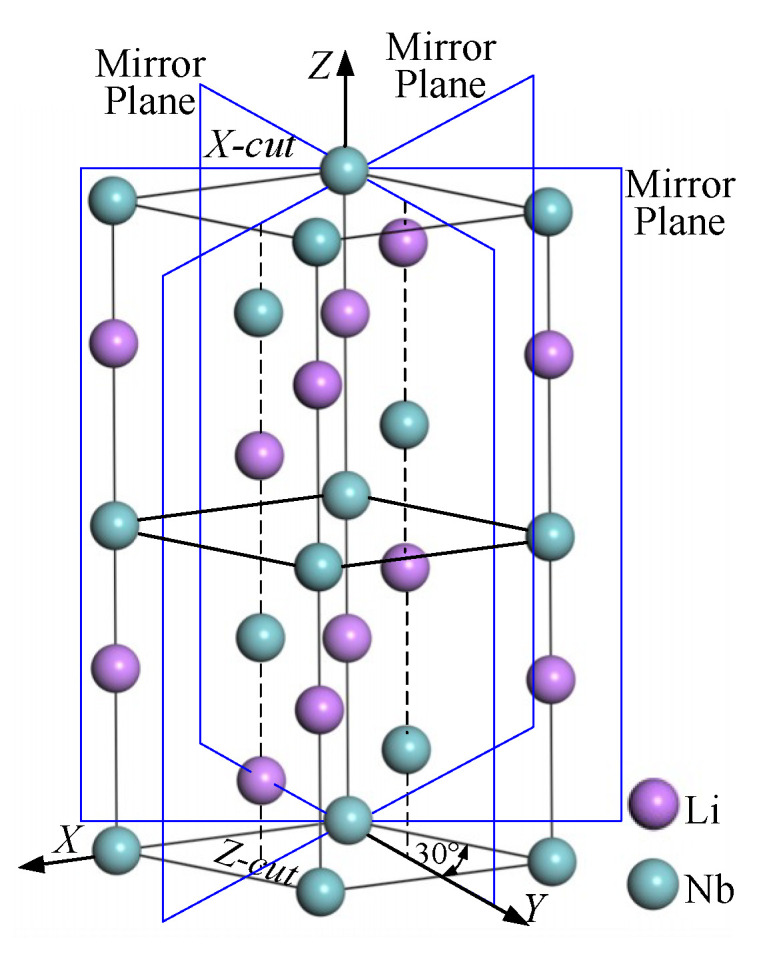
Slip orientations (X axis) in LN cell unit.

Plastic deformation and slip tend to happen when the shear stress along three X axis orientations (with a larger *d_c_* on Z-cut) [23]. There are six equivalent orientations on Z-cut because of three mirror planes (shown in Figure 11), while Y axis locates in one of the mirror planes. By the relative positions shown in Figure 11, it can be seen that the Y axis deviates the farthest from X axis and the angles between other orientations and the X axis are all less than 30°. So, the dislocation is difficult to slip along the Y-axis since it deviates far away from the slip orientation and the material removal mode tends to be brittle. Judged by the orientations relationship between the X axis and Y axis on Z-cut, the *d*_c_ along X axis is the maximum and that along Y axis the minimum. On X-cut, Y axis inclines at an angle of 30° with slip orientation X axis and Z axis is always perpendicular to glide orientation. The *d*_c_ along Z axis is the minimum in this crystal and the material removal mode along the Z axis tends to be brittle.

The bonding strength between atoms and dislocation slip systems affects the anisotropy of mechanical properties of LN wafers on different planes and orientations. Based on the arrangement of Li atoms and Nb atoms in the LN cell unit (shown in Figure 11), the Z-cut plane is regarded as the slip plane and atoms on this plane are arranged closest with minimal atom space, resulting in the strongest inter-atomic bonding force. While the space between the adjacent planes on Z-cut and are the largest, causing poor adhesion and easy to slip. When the shear force acts along the Y-axis of X-cut, the Z-cut plane shares part of the force because of the sliding tendency, leading to the deformation on the Y-axis correspondingly slighter. Therefore, the *d*_c_ of the Y-axis on X-cut is larger. The atoms are arranged looser on X-cut and the space between planes is smaller. It’s difficult to slip due to the little inter-planar space and strong bonding force. When the shear force acts along the Y-axis of Z-cut, the shear force acts almost entirely on the Y-axis for the high slip resistance on the X-cut. The Y-axis is prone to brittle fracture, resulting from the measured *d*_c_ value along the Y-axis on Z-cut being slightly less than that of the Y-axis on X-cut.

### 3.3. The MRR and Ra of X-Cut and Z-Cut

The material removal mechanism of fixed-abrasive lapping is mainly two-body ductility removal and two-body brittle fracture removal. Slight three-body rolling removal appears by dull abrasive grains falling and rolling between the pad and workpiece surface. The MRR and Ra mainly depend on the cutting depth and the ratio of material brittleness/plastic removal. The MRR and Ra increases with the cutting depth and the brittleness removal increasing. On the contrary, when the shallow cutting depth and the plastic removal are dominant, the MRR and Ra decrease.

**Figure 12:**
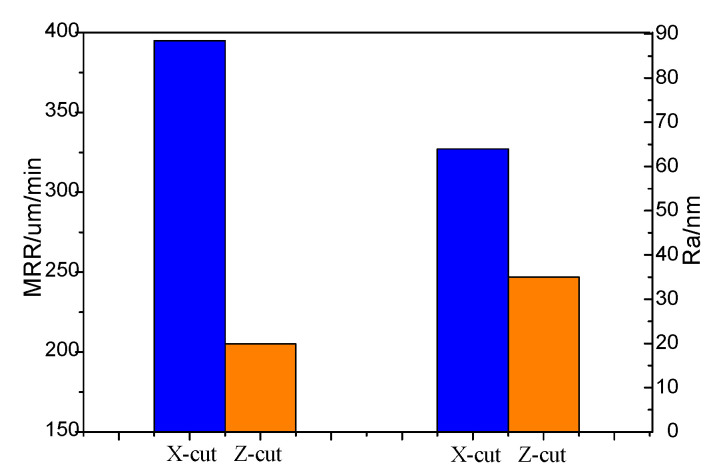
MRR and Ra of Z-cut and X-cut.

**Figure 13:**
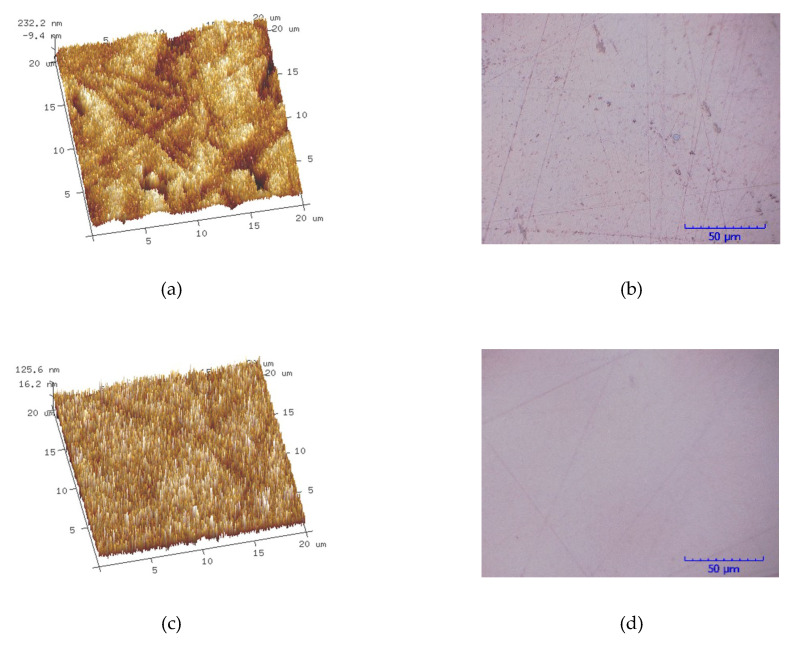
Ra and surface macro-morphology of X-cut and Z-cut. (**a**) Ra of X-cut (Ra 64 nm); (**b**) surface morphology of X-cut; (**c**) Ra of Z-cut (Ra 35 nm); (**d**) surface morphology of Z-cut.

The MRR and Ra of X-cut and Z-Cut under the same lapping conditions are shown in Figure 12. The *H*_I_ of X-cut is slightly lower than that of Z-cut. Therefore, the abrasives are easier cutting into the X-cut wafer surface under the same applied load. Due to the low *E* of X-cut and poor deformation resistance ability, the material is prone to be separated from the surface by ploughing. In addition, the *d*_c_ of X-cut is generally less than that of Z-cut, causing a greater brittleness removal proportion. These factors promote the increasing of the MRR of X-cut. Simultaneously, the high brittle removal rate results in the Ra of X-cut greater than that of Z-cut. Figure 13 shows the Ra and the surface morphology of X-cut and Z-cut. The brittle removal pits are full of the X-cut surface and the scratches are deeper. Meanwhile, there are few brittle removal traces on the Z-cut surface, and the scratches are finer. Because of the higher plastic removal ratio, the MRR of Z-cut was lower and the surface of Z-cut was smoother.

## 4. Conclusions

In this study, the nano-indentation and nano-scratching tests were conducted on LN X-cut and Z-cut wafers. The effect of anisotropy on the lapping process was explored. The following conclusions can be drawn.

(1) The *H*_I_ on Z-cut is slightly higher than that of X-cut, while the *E* of Z-cut is about 1.1 times that of the latter.

(2) The *d*_c_ varies from orientations. The *d_c_* of X axis on Z-cut is the greatest, and that of Z axis on X-cut is the smallest.

(3) The *d_c_* of Y axis on X-cut is slightly higher than that on Z-cut.

(4) With the same lapping conditions, the MRR of X-cut is higher, while the Ra of Z-cut is lower.

The anisotropic mechanical properties can be contributed to select the machining orientation in lapping process. High material removal efficiency is expected during the rough lapping process, with tolerable surface quality. When the process parameters are selected, the abrasive grain cutting depth can be designed to be greater than the maximum *d*_c_ value on the crystal plane. Therefore, the removal rate will be high because of a high percentage of the brittle removal on the workpiece surface. In reverse, high surface quality is expected during the fine polish process, with a low material removal rate. The cutting depth should be less than the minimum *d*_c_ value on the workpiece surface, resulting in the large proportion of ductile removal. For the same reason, the process parameters in fine lapping and rough polishing should result in a cutting depth between the maximum *d*_c_ and the minimum *d*_c_ values. Quantitative evaluation of the anisotropic mechanical properties is beneficial to determine the process parameters in the lapping and polishing process. This method can be also applied to other anisotropic soft-brittle crystals, by evaluating the anisotropic mechanical properties and then determining the corresponding lapping and polishing process parameters.

## Figures and Tables

**Table 1:** Brittle-ductile transition critical depth and critical load of different orientations.

Scratch Orientation		Transformation of Stage II-III/μm	*d*_c_/nm	*F*_c_/mN
Z-cut	X axis	91.0	117.5	11.650
	Y axis	60.4	66.3	7.878
X-cut	Y axis	75.8	77.4	9.787
	Z axis	44.0	36.2	5.888
